# Time to move? Factors associated with burden of care among informal caregivers of cognitively impaired older people facing housing decisions: secondary analysis of a cluster randomized trial

**DOI:** 10.1186/s12877-019-1249-1

**Published:** 2019-09-09

**Authors:** Alexandrine Boucher, Julie Haesebaert, Adriana Freitas, Rhéda Adekpedjou, Marjolaine Landry, Henriette Bourassa, Dawn Stacey, Jordie Croteau, Painchaud-Guérard Geneviève, France Légaré

**Affiliations:** 1 Canada Research Chair in Shared Decision Making and Knowledge Translation, and Population Health and Practice-Changing Research Group, Université Laval Primary Care Research Centre (CERSSPL-UL), Quebec, Canada; 2 Department of Family Medicine and Emergency Medicine, Faculty of Medicine, Université Laval, Quebec, Canada; 3 Department of Nursing of Université du Québec à Trois-Rivières, Quebec, Canada; 4 Caregiver Partner, Quebec, Canada; 5 Ottawa Hospital Research Institute, Ottawa, Canada; 6 School of Nursing, University of Ottawa, Ottawa, Canada; 7 Centre intégré universitaire de santé et services sociaux (CIUSSS) de la Capitale-Nationale, Pavillon Landry-Poulin, entrée A-1-2, bureau A-4574, 2525, chemin de la Canardière, Quebec, QC G1J 0A4 Canada

**Keywords:** Caregiver, Burden of care, Cognitively impaired older persons, Housing decisions, Shared decision making, Secondary analysis

## Abstract

**Background:**

Making health-related decisions about loved ones with cognitive impairment may contribute to caregiver burden of care. We sought to explore factors associated with burden of care among informal caregivers who had made housing decisions on behalf of a cognitively impaired older person.

**Methods:**

We conducted a secondary analysis within a cluster randomized trial (cRT) conducted in 16 publicly-funded home care service points across the Province of Quebec. The cRT assessed the impact of training home care teams in interprofessional shared decision making (IP-SDM). We assessed burden of care with the Zarit Burden Interview (ZBI) scale. We adapted Pallett’s framework to inform our data analysis. This framework posits that factors influencing burden of care among caregivers fall within four domains: (a) characteristics of the caregiver, (b) characteristics of the cognitively impaired older person, (c) characteristics of the relationship between the caregiver and the cognitively impaired older person, and (d) the caregiver’s perception of their social support resources. We computed the ZBI score and performed multilevel linear regression modelling.

**Results:**

Among 296 caregivers included in the dataset, the mean ZBI score was 29.8 (SD = 17.5) out of 88. The typical participant was 62.6 years old (SD = 11.7), female **(**74.7%), and caring for a mother or father (61.2%). Using multivariate analysis, factors significantly associated with caregiver burden mapped onto: caregiver characteristics (caregivers with higher burden were female, experienced higher decision regret and decisional conflict, preferred that their loved one move into the caregiver’s home, into a private nursing home or a mixed private-public nursing home, and had made the decision more recently); relationship characteristics (spouses and children experienced higher burden); and caregiver’s perception of social support resources (caregivers who perceived that a joint decision making process had occurred had higher burden).

**Conclusion:**

In line with the proposed framework used, we found that caregiver characteristics, relationship characteristics and caregiver’s perception of social support resources were associated with burden of care. Our results will help design interventions to prevent and/or reduce caregivers’ burden of care.

**Trial registration:**

NCT02244359. Date of registration: September 18, 2014.

## Background

Projections made in 2017 estimate that the world population aged 60 or above will more than double and the population aged over 80 will triple by 2050 [1]. Most very old people (85+) are still living at home, mostly because they want to [2–5]. However, all older people, wherever they are living, eventually have difficulty remaining independent and require assistance. Loss of autonomy among older people is commonly compensated by spouses and children [6, 7]. When the older person is cognitively impaired, formal caregivers (e.g. home care personnel) and/or informal caregivers (e.g. a family member) are often involved in decision-making about their health [8, 9]. These informal caregivers have a crucial role in ensuring the quality of life of cognitively impaired older people and in decisions related to their health. This role may entail financial burden, social isolation, and physical and/or mental health problems, referred to as “burden of care” [10].

When it is no longer possible to receive the care they need at home, cognitively impaired older people (and their caregivers) face a decision about staying at home or moving to a long-term care facility [11]. Although cognitively impaired people may benefit from participating in the decision-making process [12], caregivers often have to make the final decision on behalf of their loved one. For caregivers, making this housing decision on behalf of a cognitively impaired older person can be one of the most stressful situations [13]. Caregivers need decision support but this is not often available from healthcare professionals and caregivers frequently feel abandoned or unrecognized by the healthcare system [14, 15].

Many studies on burden of care among caregivers of people with cognitive impairment inform us about the health of caregivers. Indeed, caregiver health can affect the quality of the care they are providing and the decisions they make for the person in their care [16, 17]. Caregiver burden itself is associated with institutionalization of the care recipient, worsening of their behavioral and psychological symptoms of dementia, elder abuse, deterioration of cognition and mood, deterioration of quality of life and function, malnutrition and feeding difficulties, and increased healthcare utilization and cost of care [18]. Some studies have explored factors contributing to caregiver burden, such as characteristics of the caregivers, their preferences and values, and support available for them [9, 11, 19, 20]. In terms of housing decisions, some studies have explored caregivers’ contribution to the decision to move a cognitively impaired older person out of his/her own home [6, 9, 15]. Others have shown that this decision can increase burden of care for caregivers [9, 11, 21]. But few studies have explored risk factors for burden of care among caregivers making this decision.

## Methods

### Study design and setting

We performed a secondary analysis of data collected between March 2015 and September 2016 in 16 distinct communities in the Province of Quebec, Canada, for a two-arm cluster randomized trial (cRT) [22]. This cRT assessed the impact of training home-care teams based in community health and social services centres (HSSCs) in interprofessional shared decision making (IP-SDM) on the role that caregivers assume in the decision-making process regarding housing for a cognitively impaired older person. The intervention arm received training in IP-SDM (including the use of a decision-making guide [23]) while the control arm continued to provide usual care. The published study protocol provides complete details [22]. Below we summarize the methods relevant to this sub-analysis. We chose to treat the whole dataset as a cross-sectional study and exposure to the intervention was treated as an independent variable at the level of the cluster, namely the HSSC.

### Study participants

Participants of the primary study were a) HSSCs located in both urban and rural settings in the Province of Quebec, b) home care teams (one per HSSC), and c) informal caregivers of a cognitively impaired older person. A detailed description of the HSSC recruitment process is available elsewhere [24]. The 16 recruited HSSCs were mostly rural (67.7%) [25]. Caregivers were recruited consecutively if they were: i) involved in making a housing decision (in the months following the intervention) on behalf of a cognitively impaired person aged 65 years or older who was receiving services from an interprofessional home care team participating in the study; and ii) able to read, understand and write French or English. Therefore all caregivers from both the intervention and control groups were included in this secondary analysis, representing a total of 296 caregivers of cognitively impaired older persons receiving care from interprofessional home care teams in the participating HSSCs.

### Data collection

After obtaining the caregiver’s written informed consent, a trained research assistant completed the study questionnaire with the caregiver in a place chosen by the participant (either in a public place, such as a café, or in the caregiver’s home). Participation of the caregiver was cross-sectional, i.e. there was only one data collection point for each participant. In total, 27 variables were available in the dataset of the parent study. Our approach to selecting the independent variables from this dataset was informed by Pallett’s (1990) conceptual framework for assessing caregivers’ burden of care in Alzheimer’s dementia [20]. This framework posits that factors affecting burden of care fall under four broad domains a) characteristics of the caregiver (e.g., sociodemographics, values and preferences about the healthcare of the older person, health and emotional condition); b) characteristics of the cognitively impaired older person (e.g., cognitive impairment, behavior problems, values and preferences about their healthcare, and type and amount of care required); c) characteristics of the relationship between the caregiver and the cognitively impaired older person (e.g., quality and relationship type); and d) the caregiver’s perception of available social support resources (e.g., social networks, family relationships, and support from healthcare professionals). Variables within each domain may act either to induce caregiver burden of care, or to protect the caregiver against it. We adapted Pallett’s framework to our context of caregivers who had made housing decisions on behalf of a cognitively impaired older person and mapped our dependent and independent variables onto its four domains to ensure our secondary analysis was theory informed.

### Caregiver burden of care

Our dependent variable was assessed using the Zarit Burden Interview (ZBI) scale [10]. The ZBI is a validated scale consisting of 22 items, each of which is measured on a five-point Likert scale (0 = never, to 4 = nearly always) and a total score ranging from 0 (no burden) to 88 (severe burden) [10, 26–28]. Internal consistency of the scale is good to excellent (Cronbach alpha correlation coefficients range from 0.85 to 0.93) [26, 29]. The test-retest reliability is high (intra-class correlation coefficient 0.89) [26, 29].

### Independent variables

*Decision regret* was assessed with the 5-item Decision Regret Scale (DRS) [30, 31]. The score of each item was converted to a scale of 0 (no regret) to 100 (high regret), in increments of 5.

*Decisional conflict*, or personal uncertainty about the course of action to take when the choices involve risk, loss, regret, or a challenge to personal life values, was measured with the 16-item Decisional Conflict Scale (DCS) [32, 33]. This scale also captures factors contributing to uncertainty, such as feeling uninformed, being unclear about personal values, feeling unsupported and perception of making an effective decision. Scores range from 0 (no decisional conflict) to 100 (high decisional conflict).

*The caregiver’s perception of the role they assumed in decision-making* was measured using a modified version of the Control Preferences Scale, a single question that assesses perception of locus of control over decision-making [34, 35]. Response options are: A) I made the decision, B) I made the decision after considering the healthcare professionals’ opinions, C) The healthcare professionals and I shared the responsibility for the decision-making, D) The healthcare professionals made the decision after considering my opinion, E) The healthcare professionals made the decision. Based on previous work in this field, we dichotomized these into an active role (A, B, C) or a passive role (D and E) [35].

*The caregiver’s perception that a joint decision-making process had occurred with a member or members of the home care team* was measured using the Dyadic Observing Patient Involvement in Decision Making instrument (D-OPTION) [36, 37]. This 12-item instrument assesses the caregiver’s perception that a joint decision-making process occurred and the final score ranges from 0 (decision-making not joint) to 100 (decision-making joint).

*Caregivers’ perceptions of healthcare professionals’ SDM behaviors during the decision-making process* was measured with the 9-item Shared Decision Making Questionnaire (SDMQ-9) rated on a 6-point scale [38]. The score ranges from 0 to 100, where 0 is the absence of any SDM behaviors and 100 indicates the presence of all nine.

### Mapping the independent variables

Of the 27 independent variables available in our dataset, we mapped 21 onto Pallett’s conceptual framework. The six other variables related to the fidelity of the intervention and we not appropriate for our analysis.

#### Characteristics of the caregiver

We mapped 13 variables onto this domain, including sociodemographic characteristics (age, sex, education level, total family income, civil status, and employment status) as well as two variables relating to the caregiver’s values and preferences. The latter were what role he/she wished to play in the decision-making process (preferred role), and his/her housing preference for the cognitively impaired older person (stay home, move in with caregiver, move to private or public nursing home or another option). This domain also included decisional conflict (DCS) and decision regret about the actual decision made (DRS). Variables relating to the caregiver’s emotional reaction to the decisional process and its evolution with time were also included in this domain. We also mapped the number of days that had elapsed between when the caregiver made the decision and when they completed the questionnaire. The main RCT used post-intervention measures, so caregivers made the decision after their home care team had received the intervention. The questionnaires were administered once the home care teams had identified caregivers who had made a decision since the intervention, so the time lapse varied between the decision and the questionnaire (we refer to this henceforth as “time lapse since the decision.”) The season when the housing decision had to be made, and the season when the caregiver entered the study were mapped onto this domain because they could influence the caregiver’s emotional reaction which in turn could influence burden of care [17]. Quebec has extreme weather conditions, varying from very hot in summer (> 30 C) to extremely cold in winter (< 30 C) and this may influence their perception of the built environment as being adequate or not during these conditions.

#### Characteristics of the cognitively impaired older person

We mapped two variables regarding the housing decision onto this domain: the cognitively impaired person’s housing preference, according to the caregiver, and the actual housing decision made, i.e. where the cognitively impaired older person would live in the future.

#### Characteristics of the relationship between the caregiver and the cognitively impaired older person

We mapped one variable onto this domain: the relationship type between caregiver and the cognitively impaired older person, i.e. if they were taking care of a spouse, a mother or father, a friend, another family member, or other.

#### The caregiver’s perception of available social support resources

We mapped four variables onto this domain. Three of them informed us about the extent to which caregivers felt engaged and supported in their decision: the caregiver’s perception of the role he or she had assumed in the decision-making process, i.e. passive or active (Control Preferences Scale); the caregiver’s perception that a joint decision-making process with a home care team member had occurred (D-OPTION); and caregivers’ perceptions of healthcare professionals’ SDM behaviors during the decision-making process (SDMQ-9). Although related, these three measures capture distinct concepts. The fourth variable we mapped onto this domain was whether the caregiver was part of the intervention group or the control arm in the main study, i.e. whether or not their home care team had received (been exposed to) the IP-SDM intervention [22]. Finally, we noted whether the HSSC was in a rural or urban setting.

### Data analysis

We calculated percentages, means and standard deviations to describe the population of caregivers. All variables had less than 1% missing values. For missing values, we imputed by the individual mean imputation method or using a strongly correlated variable when possible. We imputed the value of the scores by the mean of the subject’s complete responses to other items [39]. We categorized the DRS score to facilitate interpretation because of the high percentage score of 0. Based on results of a systematic review of 59 studies reporting on decision regret, we recoded it as: “no regret” (DRS score < 5), “mild regret” (5 ≤ DRS score ≤ 25) and “moderate to strong regret” (DRS score ≥ 30) [31].

First, we explored potential independent variables associated with ZBI by performing unadjusted analyses using two-level simple regression (HSSCs and caregivers), including the HSSCs as a random effect at the level of the intercept in order to account for the intra-class correlation induced by the HSSCs. Before running the multivariate model, we tested possible mediators in order to identify potential variables involved in the same causal path thereby avoiding their simultaneous inclusion in the multivariate analysis. The multiple regression model included all potential associated variables identified with the four domains of the adapted Pallett’s framework as described above. We built a final parsimonious theory-informed model by including all predictors retained by a backward selection procedure with *p* ≤ 0.05 as the stopping rule. When results between the bivariate and multivariate estimates were inconsistent, we tested possible interactions between independent variables. In all analyses, we verified model assumptions such as normality and homoscedasticity of residuals. We estimated the intra-class correlation coefficients (ICC) for the two-level unadjusted model. For a sample size of 296 caregivers, the estimated power of our analysis was 86% considering a two-level multiple regression analysis with 16 clusters, an ICC of 0.1 and a moderate effect size of 0.15. The analyses were performed using R version 3.4.3.

## Results

### Caregiver burden of care

All 296 caregivers included in the parent study were included in our analysis. On average, caregivers’ ZBI mean ± standard deviation score (SD) was 29.8 ± 17.5 out of 88. The results of the ZBI score ranged from 0 to a maximum of 81. ICC for the ZBI was 0.13 (Table 1).

**Table 1:** Caregiver burden of care and independent variables mapped onto an adaptation of Pallett’s conceptual framework

Variables	Caregivers (*n* = 296)
*Variable of interest*	
Caregiver’s burden of care^a^, mean (SD)	29.8 (17.5)
Score range (minimum-maximum)	0–81
*Independent variables*	
**Caregiver’s characteristics**	
Age, mean (SD)	62.6 (11.7)
Score range (maximum-minimum)	33.1–97.9
Sex, *n* (%)	
Female	221 (74.7)
Male	75 (25.3)
Civil status, *n* (%)	
Married/Common-law partner	230 (77.7)
Single	30 (10.1)
Separated/Divorced	22 (7.4)
Widower	14 (4.7)
Employment status, *n* (%)	
Retired	149 (50.3)
Employed	118 (39.9)
At home	22 (7.3)
Unemployed/ Job seeker	7 (2.4)
Education level, *n* (%)	
Primary	30 (10.1)
Secondary	135 (45.6)
College	67 (22.6)
University	64 (21.6)
Total family income, *n* (%)	
< 15,000	17 (5.7)
15,000–29,999	52 (17.6)
30,000–44,999	43 (14.5)
45,000–59,999	53 (17.9)
60,000 and more	77 (26.0)
Prefered to not answer	54 (18.2)
Caregiver preferred role in the decision-making process, *n* (%)	
A) I want to make the decision alone	30 (10.1)
B) I want to make the decision after considering the healthcare professionals’ opinions	105 (35.5)
C) I want the healthcare professionals and I to share the responsibility for the decision-making	113 (38.2)
D) I want the healthcare professionals to make the decision after considering my opinion	44 (14.9)
E) I want that the healthcare professionals to make the decision alone	3 (1.0)
Missing	1 (0.3)
Caregiver’s housing preference for the cognitively impaired older person, *n* (%)	
Public nursing home	45 (15.2)
Stay at home	128 (43.2)
Caregiver’s home	24 (8.1)
Private nursing home	84 (28.4)
Other	15 (5.1)
Decisional regret^b^, *n* (%)	
(< 5)	171 (57.8)
(≥5 à ≤25)	80 (27.0)
(≥30)	45 (15.2)
Decisional conflict^c^, mean (SD)	23.3 (17.6)
Decisional conflict subscales ***	
Informed subscale, mean (SD)	27.6 (24.7)
Values clarity subscale, mean (SD)	19.8 (18.3)
Support subscale, mean (SD)	26.0 (22.6)
Uncertainty subscale, mean (SD)	27.6 (22.4)
Effective decision subscale, mean (SD)	17.5 (19.2)
Time lapse (days) since the decision was made, mean (SD)	142.6 (104.9)
Season when the questionnaire was completed by caregivers, *n* (%)	
Winter	76 (25.7)
Spring	144 (48.6)
Summer	44 (14.9)
Fall	32 (10.8)
Season when the decision had to be made, *n* (%)	
Winter	78 (26.4)
Spring	72 (24.3)
Summer	67 (22.6)
Fall	79 (26.7)
**Characteristics of cognitively impaired older person**	
Cognitively impaired older person’s housing preference, according to the caregiver, *n* (%)	
Public nursing home	17 (5.7)
Stay at home	184 (62.2)
Caregiver’s home	20 (6.8)
Private nursing home	46 (15.5)
Other	5 (1.7)
Does not apply	23 (7.8)
Missing	1 (0.3)
Actual housing decision made, *n* (%)	
Public nursing home	98 (33.1)
Stay at home	43 (14.5)
Caregiver’s home	10 (3.4)
Private nursing home	91 (30.7)
Other	54 (18.2)
**Characteristics of the relationship between the caregiver and the cognitively impaired older person**	
Relationship type between caregiver and the cognitively impaired older person, *n* (%)	
Child	181 (61.2)
Spouse	66 (22.3)
Other member of family	42 (14.2)
Friend or other	7 (2.4)
**Caregiver’s social support and resources perception**	
Caregiver’s perception of his/her assumed role in the decision making process, *n* (%)	
A) I made the decision alone	47 (15.9)
B) I made the decision after considering the healthcare professionals’ opinions	88 (29.8)
C) The healthcare professionals and I share the responsibility for the decision-making	83 (27.8)
D) The healthcare professionals made the decision after considering my opinion	57 (19.3)
E) The healthcare professionals made the decision alone	21 (7.1)
Caregiver’s perception of the occurrence of a joint decision-making process (D-OPTION)^d^, mean (SD)	63.8 (20.6)
Caregivers’ perceptions of healthcare professional’s specific SDM behaviors during the decision-making process (SDMQ-9)^e^, mean (SD)	64.9 (25.3)
Caregiver’s home care team has received SDM training *n* (%)	138 (46.6)

*SD* Standard deviation, *D-OPTION* Dyadic Observing Patient Involvement in Decision Making instrument, *SDMQ-9* 9-item Shared Decision Making Questionnaire, *IP-SDM* interprofessional shared decision making,

^a^ZBI scale, score from 0 to 88.

^b^DRS score < 5 = no regret, ≥5 to ≤25 = mild regret and ≥ 30 = moderate to strong regret [31];

^c^score from 0 (no decisional conflict) to 100 (high decisional conflict)

^d^score from 0 (decision-making not joint) to 100 (decision-making joint);

^e^for one point increase, score from 0 (no SDM behaviors) to 100 (all SDM behaviors)

### Independent variables

#### Characteristics of the informal caregiver

Mean age of caregivers was 62.6 ± 11.7 years, 74.7% were female, 50.3% were retired, and the highest level of formal education for 45.6% of caregivers was secondary school (Table 1). Close to half (43.2%) preferred that the cognitively impaired older person stay at home, and 43.6% preferred they move to a nursing home. Most caregivers experienced no decision regret (57.8%), 27.0% felt mild decision regret and 15.2% moderate to high decision regret. Mean DCS score was 23.3 ± 17.6 (scores of ≥25/100 are clinically significant [40]). Time lapse since the decision was 142.6 ± 104.9 days.

#### Characteristics of the cognitively impaired older person

The preference of 62.2% of the cognitively impaired older people, according to their caregivers, was to stay at home. Regarding the housing decision eventually made, 63.8% moved to a private or public nursing home, 14.5% stayed in their home, 3.4% moved into the caregiver’s home, and 18.2% moved to another location, such as a mixed private-public nursing home.

#### Characteristics of the relationship between the caregiver and the cognitively impaired older person

Caregivers were mostly children (61.2%) or spouses (22.3%) of the cognitively impaired older person.

#### The caregivers’ perception of available social support resources

A total of 73.5% of caregivers said they were active in decision making. The mean score of the caregiver’s perception of the occurrence of a joint decision-making process with a home care team member (D-OPTION) was 63.8 ± 20.6 out of 100. The mean score of the caregivers’ perceptions of healthcare professionals’ SDM behaviors during the decision-making process (SDMQ-9) was 64.9 ± 25.3 out of 100. Finally, 46.6% of caregivers were receiving services from a team who had received SDM training.

### Bivariate analysis

The following variables were found to be significantly associated with caregiver burden of care (Table 2): education level, caregiver’s preferred role in the decision-making process, decision regret, decisional conflict, the time lapse since the decision was made, relationship type between caregivers and the cognitively impaired older person, and the caregiver’s perception that a joint decision making process with a home care team member had occurred. Two other variables, sex and the caregiver’s housing preference for the cognitively impaired older person were narrowly statistically significant.

**Table 2:** Two-level simple regression analysis of factors associated with caregiver burden of care

Independent variables	β (95% CI)		*p*-value
**Characteristics of the caregiver**			
Age	0.08 (−0.08, 0.25)		0.31
Sex			
Men	Reference		0.07
Women	4.01 (−0.35, 8.38)		
Civil status			
Single	Reference		0.15
Married/common-law partner	−4.49 (− 10.85, 1.86)		
Separated/divorced	−7.45 (− 16.6, 1.69)		
Widower	−11.39 (− 21.89, −0.89)		
Employment status			
At home	Reference		0.22
Unemployed/ Job seeker	10.74 (−3.26, 24.73)		
Retired	3.66 (−3.9, 11.23)		
Employed	0.65 (−6.96, 8.27)		
Education level^a^			
Secondary	Reference		0.02
Primary	0.13 (−6.72, 6.97)		
College	−6.14 (−10.44, −1.83)		
University	−0.55 (−5.64, 4.54)		
Total family income^a^			
Less than 15,000	Reference		0.15
15,000–29,999	0 (−10.57, 10.58)		
30,000–44,999	−3.53 (− 14.46, 7.4)		
45,000–59,999	− 5.2 (− 15.68, 5.28)		
60,000 and more	−6.61 (− 16.76, 3.55)		
Preferred to not answer	−1.18 (− 12.21, 9.86)		
Caregiver’s preferred role in the decision-making process			
Passive role	Reference		0.04
Active role	−5.64 (−10.93, −0.34)		
Caregiver’s housing preference for the cognitively impaired older person			
Public nursing home	Reference		0.05
Stay at home	7.27 (1.59, 12.95)		
Caregiver’s home	9.62 (1.49, 17.74)		
Private nursing home	7.94 (1.96, 13.92)		
Other	9.73 (−0.06, 19.53)		
Decision regret^b^			
(< 5)	Reference		< 0.001
(≥5 to ≤25)	5.68 (1.36, 10.01)		
(≥30)	10.97 (5.54, 16.41)		
Decisional conflict^c^	0.13 (0.02, 0.24)		0.02
Decisional conflict subscales			
Informed subscale	0.05 (−0.03, 0.13)		0.26
Values clarity subscale	0.09 (−0.01, 0.20)		0.09
Support subscale	0.04 (−0.04, 0.13)		0.30
Uncertainty subscale	0.14 (0.06, 0.23)		0.001
Effective decision subscale	0.12 (0.02, 0.22)		0.02
Time lapse (days) since the decision was made^c^	−0.02 (−0.04, 0)		0.02
The season when the housing decision had to be made			
Winter	Reference		0.17
Spring	−2.1 (−7.49, 3.28)		
Summer	−6.06 (−11.53, −0.58)		
Fall	−1.76 (−6.99, 3.48)		
The season when the caregivers were entered the study			
Winter	Reference		0.35
Spring	−3.75 (−8.86, 1.35)		
Summer	−4.65 (−11.52, 2.22)		
Fall	−5.03 (−12.04, 1.99)		
**Characteristics of the cognitively impaired older person**			
Cognitively impaired older person’s housing preference, according to the caregiver^a^			
Public nursing home	Reference		0.10
Stay at home	8.05 (1.08, 15.03)		
Caregiver’s home	14.27 (4.34, 24.19)		
Private nursing home	6.51 (−1.5, 14.51)		
Other	8.21 (−6.79, 23.22)		
Does not apply	11.15 (−0.08, 22.37)		
The actual health related housing decision made by the caregiver for the cognitively impaired older person			
Public nursing home	Reference		0.36
Stay at home	−1.33 (−7.58, 4.93)		
Caregiver’s home	5.67 (−5.33, 16.67)		
Private nursing home	2.92 (−2.12, 7.97)		
Other	4.09 (−1.65, 9.82)		
**Characteristics of the relationship between the caregiver and cognitively impaired older person**			
Relationship type between caregiver and the cognitively impaired older person^a^			
Other family member	Reference		< 0.001
Child	10.21 (6.16, 14.25)		
Friend or other	7.06 (0.08, 14.04)		
Spouse	19.19 (13.72, 24.66)		
**Caregiver’s perception of social support resources**			
Caregiver’s assumed role in the decision-making process			
Passive role	Reference		0.90
Active role	−0.3 (−4.74, 4.15)		
Caregiver’s perception of the occurrence of a joint process in the decision-making (D-OPTION)^d^	0.11 (0.01, 0.20)		0.02
Caregivers’ perceptions of healthcare professional’s SDM behaviors during the decision-making process (SDMQ-9)^c^	0.02 (−0.05, 0.1)		0.52
Caregiver’s home care team has received SDM training			
Yes	Reference		0.60
No	−2.04 (−10.27, 6.19)		
Health and social services centre (HSSC) setting			
Urban/suburban	Reference		0.11
Rural	−6.69 (−15.08, 1.71)		

*D-OPTION* Dyadic Observing Patient Involvement in Decision Making instrument, *SDMQ-9* 9-item Shared Decision Making Questionnaire, *IP-SDM* interprofessional shared decision making*, HSSCs* health and social services centres

^a^Model not assuming equal variance

^b^DRS score < 5 = no regret, ≥5 to ≤25 = mild regret and ≥ 30 = moderate to strong regret [31]

^c^for one point increase

^d^for one point increase, score from 0 = decision-making not joint to 100 = decision-making joint

### Multivariate analysis

Overall, our final model explained 39% of the total variance of the dependent variable, with the cluster (HSSC) explaining 13% alone (Table 3). At the level of the caregiver, the following variables were significantly associated with caregiver burden: sex (women were more likely to experience higher burden), caregiver’s housing preference for the cognitively impaired older person (preference that the person move into their own home, into a private nursing home or a mixed private-public intermediate facility was associated with more burden), decision regret (more decision regret was associated with more burden), decisional conflict (more decisional conflict was associated with more burden), time lapse since the decision was made (more time was associated with less burden), relationship type (being a spouse of the person cared for was associated with more burden), and caregivers’ perception of a joint decision making process (i.e., regardless of what the caregiver’s preferred role was, the more they perceived that a joint decision making process had occurred, the higher the burden of care was). In this analysis, none of the factors in the domain of the cognitively impaired person’s characteristics remained significantly associated with higher burden of care.

**Table 3:** Two-level multiple regression analysis of factors associated with caregiver’s burden of care

Cluster-level (HSSCs random effect at intercept level: ICC = 13%)		
Caregiver-level (Marginal *R*^2^ = 26%)^a^		
Independent variables	β (95% CI)	*p*-value
**Characteristics of the caregiver**		
Sex		
Men	Reference	0.04
Women	4.15 (0.30, 7.99)	
Caregiver’s housing preference for the cognitively impaired older person		
Public nursing home	Reference	0.01
Stay at home	3.5 (−1.59, 8.59)	
Caregiver’s home	9.18 (1.93, 16.44)	
Private retirement residence	7.04 (1.76, 12.32)	
Other (e.g. mixed private-public nursing homes)	11.22 (2.68, 19.77)	
Time lapse (days) since the decision was made	−0.02 (−0.04, 0)	0.03
Decision regret^b^		
(< 5)	Reference	< 0.001
(≥5 à ≤25)	4.73 (0.76, 8.69)	
(≥30)	10.7 (5.07, 16.33)	
Decisional conflict^c^	0.15 (0.03, 0.28)	0.02
**Characteristics of the relationship between the caregiver and cognitively impaired older person**		
Relationship link between caregiver and the cognitively impaired older person		
Other family member	Reference	< 0.001
Child	7.51 (2.56, 12.46)	
Friend or other	6.78 (−5.09, 18.65)	
Spouse	17.69 (11.83, 23.54)	
**Caregiver’s social support resources perception**		
The caregiver’s perception of the occurrence of a joint process in the decision-making (D-OPTION)^d^	0.26 (0.17, 0.36)	< 0.001

*HSSCs*: health and social services centres; *ICC*: intra-class correlation coefficients; *D-OPTION*: Dyadic Observing Patient Involvement in Decision Making instrument

^a^Total variance explained by the model = 39%

^b^Decisional conflict score < 5 = no regret, ≥5 to ≤25 = mild regret and ≥ 30 = moderate to strong regret [31]

^c^For one point increase score from 0 (no decisional conflict) to 100 (high decisional conflict)

^d^for one point increase, score from 0 = decision-making not joint to 100 = decision-making joint

## Discussion

Our study evaluated factors associated with burden of care among caregivers who had made housing decisions on behalf of a cognitively impaired older person. We observed that the domains proposed by our adapted conceptual framework explained a significant proportion of the variance of burden of care, thus providing further evidence to validate the framework. We found six risk factors for higher burden of care and one for lower burden of care. These results lead us to make three main observations.

First, our mean score of caregiver burden was slightly lower than in a set of five other studies on burden of care among caregivers of cognitively impaired older people [41–45]. Our study population was receiving services from interprofessional home care teams and may have been experiencing less burden because of this support. Also, given that some of them had experienced the housing decision at least 3 months before the data collection, it is possible that our participating caregivers had time to find various strategies to alleviate their burden of care [9]. However, other results in our sample are congruent with other studies: caregivers were mostly female, in their mid-sixties, and taking care of a mother or father [7, 15].

Second, we observed that most of the factors associated with burden of care were congruent with findings of other studies in this field. For example, we observed that women experienced more caregiver burden than men, and spouses more than children of the cognitively impaired older person. Women, especially spouses and daughters, generally take on the role of caregivers [6, 8]. Indeed, for gendered cultural reasons, many women believe they are responsible for caring because of their sex. Female spouses may in addition believe that caring is part of their duty as a partner [14, 17] and as they have often cared more directly for children they have more experience of home care than men [46, 47]. Taking care of a mother/father was associated with significant caregiver burden, a result which has been found elsewhere [17]. Children of a cognitively impaired older person may have less experience in caregiving, which may increase the difficulty of their caregiving responsibilities [6, 7, 17]. Also, they are more likely to be trying to balance the demands of caring for parents, caring for their own children, and working full-time, and they may feel that they can’t give enough time to caring [6, 7, 17]. Our observation about decision regret and decisional conflict being associated with higher burden of care was also congruent with the literature [9, 14, 19]. Caregivers want to honor the preference of their loved one [14] and if the caregiver’s choice is not consistent with the preference of the cognitively impaired older person, it increases decision regret and decisional conflict, which in turn appears to increase the general burden on the caregiver [9].

Third, we found new factors associated with burden of care that have not yet been observed in the literature. Burden of care was higher when a caregiver’s housing preference was a private nursing home, a mixed private-public nursing home, or having the cognitively impaired person move into their own home (compared to them moving into a public, nursing home). In Quebec, private nursing homes are certified by the government but managed by private interests. Public nursing homes are managed by the state and free for eligible citizens. Mixed private/public nursing homes are private nursing homes contracted by the state to provide services for eligible residents. This higher burden of care can probably be explained by the apprehension of the high cost of sustaining a family member in a private nursing home for the rest of their life, and perhaps at a great distance from their own home, or the difficulty and upheaval of looking after a cognitively impaired person at home [6, 15]. Also, our results show that the more time that had elapsed after the decision, the less burden the caregiver experienced. Few SDM studies have considered the effects of decision-making over the long-term, beyond the immediate consequences [48]. In the short term, this difficult decision may be an emotional burden [9], and caregiver burden does not always end after institutionalization. With time, however, the caregiver may experience the institutionalization of their cognitively impaired relative as a relief [17]. The perception that they are receiving better care than before may also be the best reassurance for caregivers that they made the best decision and therefore decreases their burden of care [21]. Also, as reported by previous decision making research, people are generally bad at forecasting, so caregivers’ worst fears may have been unfounded [49]. Our results also showed that the more a caregiver perceived that a joint decision-making process had occurred with the home care team, the higher their burden of care was. This is in contrast with previous work that showed that sharing decisions with healthcare professionals may facilitate the decision-making process and decreases caregiver burden [9, 11, 14]. However, involvement in a housing decision for a loved one may also result in emotional stress due to the need to understand and process complex information [9]. This may undermine a surrogate’s ability to make decisions that protect their loved one’s interests and promote their preferences. In addition, some caregivers may prefer not take the responsibility for a decision that might require they go against the preferences and values of their loved one or indeed their own desires [12]. Some caregivers might also find it stressful and intimidating to partake in a collective decision-making process with a team of professionals [50]. Interestingly, two related variables, caregiver’s assumed role and caregiver’s perception of healthcare professional’s SDM behaviors, were not kept in the final model, suggesting that these three concept, (joint process, assumed role and perception of the health professional’s SDM behaviors) and their respective measures are clearly distinct.

### Limitations

Our study has limitations. First, this is a secondary data analysis of an earlier cRT, which did not collect data on all of the concepts in Pallett’s domains. While Pallett’s framework describes many of the factors associated with burden of care in our context, it included numerous additional factors that were not in our dataset, such as the cognitively impaired older person’s diseases and behavioral problems and the quality of the relationship between the caregiver and the cognitively impaired older person [7, 15, 17, 20]. However, the framework provided important theoretical support which resulted in a final model that successfully explained significant variance in burden of care. Second, a selection bias may have contributed to our underestimating of caregiver burden. Caregivers with too heavy a burden may have been unwilling to participate in the study because they were overwhelmed by their responsibilities. Finally, we did not measure the time elapsed since the older person had actually moved (only the time elapsed since the decision), and so we cannot affirm with certainty that the time elapsed since the decision corresponded exactly to the time elapsed since they moved.

## Conclusion

We found that burden of care in caregivers who had made housing decisions on behalf of a cognitively impaired older person was higher if caregivers were female, experienced higher decision regret and decisional conflict, and were caring for a parent. These variables map on to three of Pallet’s proposed four domains. We also found new risk factors for higher burden of care: caregivers preferring that their loved one move into their own home or into a private or mixed public/private nursing home (as opposed to a public nursing home); having experienced a joint decision-making process; and a shorter lapse of time since the decision was made.

We are unaware of any evidence-based interventions that specifically address the risk factors for burden of care identified in this paper. However, our team has developed training for home care teams in interprofessional decision-making with caregivers and their loved ones and this study challenges and informs our intervention [22]. Home care teams may be trained to identify those caregivers at higher risk of burden of care (e.g. older women facing a housing decision for a cognitively impaired parent without decision-making support) and offer adapted support services, such as psychoeducation or cognitive behavioral therapy [18]. Caregivers themselves could also be given strategies for reducing burden of care when making decisions about housing for their relatives. Even if they do not want to participate in the process, if they feel more supported this may reduce their burden [9, 11, 14].

## Data Availability

The datasets used and/or analyzed during the current study are available from the corresponding author on reasonable request.

## Abbreviations

cRT: Cluster randomized trial
DCS: Decisional Conflict Scale
D-OPTION: Dyadic Observing Patient Involvement in Decision Making instrument
DRS: Decision Regret Scale
HSSC: health and social services center
ICC: Intra-class correlation coefficients
IP-SDM: Interprofessional shared decision making
SDM: Shared decision making
SDMQ-9: the 9-item Shared Decision Making Questionnaire
ZBI: Zarit Burden Interview Scale

## References

[1] United Nations (2017). World Population Prospects. Key Findings and Advanced Tables. The 2017 Revision.

[2] Abramsson M, Andersson EK (2012). Residential mobility patterns of elderly—leaving the house for an apartment. Hous Stud.

[3] Jungers CM (2010). Leaving home: an examination of late-life relocation among older adults. J Couns Dev.

[4] Lofqvist C, Granbom M, Himmelsbach I, Iwarsson S, Oswald F, Haak M (2013). Voices on relocation and aging in place in very old age–a complex and ambivalent matter. Gerontologist.

[5] Oswald F, Wahl H-W. Creating and sustaining homelike places in residential environments. In G D Rowles & M Bernard (Eds.) Environmental gerontology: Making meaningful places in old age. New York: Springer Publishing. 2013:53–77.

[6] Chiao CY, Wu HS, Hsiao CY (2015). Caregiver burden for informal caregivers of patients with dementia: a systematic review. Int Nurs Rev.

[7] Yin T, Zhou Q, Bashford C (2002). Burden on family members: caring for frail elderly: a meta-analysis of interventions. Nurs Res.

[8] Wackerbarth S (1999). Modeling a dynamic decision process: supporting the decisions of caregivers of family members with dementia. Qual Health Res.

[9] Wendler D, Rid A (2011). Systematic review: the effect on surrogates of making treatment decisions for others. Ann Intern Med.

[10] Zarit S, Orr NK, Zarit JM (1985). The hidden victims of Alzheimer's disease: families under stress: NYU press.

[11] Ducharme F, Couture M, Lamontagne J (2012). Decision-making process of family caregivers regarding placement of a cognitively impaired elderly relative. Home Health Care Serv Q.

[12] Miller LM, Whitlatch CJ, Lyons KS (2016). Shared decision-making in dementia: a review of patient and family carer involvement. Dementia.

[13] Caron CD, Ducharme F, Griffith J (2006). Deciding on institutionalization for a relative with dementia: the Most difficult decision for caregivers. Canadian Journal on Aging / La Revue canadienne du vieillissement.

[14] Lord K, Livingston G, Cooper C (2015). A systematic review of barriers and facilitators to and interventions for proxy decision-making by family carers of people with dementia. Int Psychogeriatr.

[15] Adelman RD, Tmanova LL, Delgado D, Dion S, Lachs MS (2014). Caregiver burden: a clinical review. JAMA.

[16] Wittenberg E, Prosser LA (2016). Health as a family affair. N Engl J Med.

[17] Sansoni J, Anderson KH, Varona L, Varela G (2013). Caregivers of Alzheimer’s patients and factors influencing institutionalization of loved ones: some considerations on existing literature. Ann Ig.

[18] Stall NM, Kim SJ, Hardacre KA, Shah PS, Straus SE, Bronskill SE, et al. Association of Informal Caregiver Distress with health outcomes of community-dwelling dementia care recipients: a systematic review. J Am Geriatr Soc. 2018:67(3):609-617. 10.1111/jgs.15690 30536383

[19] Garvelink MM, Ngangue PA, Adekpedjou R, Diouf NT, Goh L, Blair L (2016). A synthesis of knowledge about caregiver decision making finds gaps in support for those who care for aging loved ones. Health Aff.

[20] Pallett PJ (1990). A conceptual framework for studying family caregiver burden in Alzheimer's-type dementia. Image J Nurs Sch.

[21] Butcher HK, Holkup PA, Park M, Maas M (2001). Thematic analysis of the experience of making a decision to place a family member with Alzheimer's disease in a special care unit. Res Nurs Health.

[22] Légaré F, Brière N, Stacey D, Bourassa H, Desroches S, Dumont S (2015). Improving decision making on location of care with the frail elderly and their caregivers (the DOLCE study): study protocol for a cluster randomized controlled trial. Trials.

[23] Garvelink MM, Emond J, Menear M, Briere N, Freitas A, Boland L (2016). Development of a decision guide to support the elderly in decision making about location of care: an iterative, user-centered design. Res Involv Engagem.

[24] Garvelink MM, Freitas A, Menear M, Brière N, Stacey D, Légaré F (2015). In for a penny, in for a pound: the effect of pre-engaging healthcare organizations on their subsequent participation in trials. BMC Res Notes.

[25] Adekpedjou R, Stacey D, Briere N, Freitas A, Garvelink MM, Turcotte S (2018). “Please listen to me”: A cross-sectional study of experiences of seniors and their caregivers making housing decisions. PLoS One.

[26] Hébert R, Bravo G, Girouard D (1993). Fidélité de la traduction française de trois instruments d'évaluation des aidants naturels de malades déments. Canadian Journal on Aging/La revue canadienne du vieillissement.

[27] Hébert R, Leclerc G, Bravo G, Girouard D, Lefrançois R (1994). Efficacy of a support group programme for care-givers of demented patients in the community: a randomized controlled trial. Arch Gerontol Geriatr.

[28] Zarit SH, Reever KE, Bach-Peterson J (1980). Relatives of the impaired elderly: correlates of feelings of burden. Gerontologist.

[29] Seng B, Luo N, Ng W, Lim J, Chionh HL, Goh J (2010). Validity and reliability of the Zarit burden interview in assessing caregiving burden. Ann Acad Med Singap.

[30] Brehaut JC, O'Connor AM, Wood TJ, Hack TF, Siminoff L, Gordon E (2003). Validation of a decision regret scale. Med Decis Mak.

[31] Becerra Perez MM, Menear M, Brehaut JC, Legare F (2016). Extent and predictors of decision regret about health care decisions: a systematic review. Med Decis Mak.

[32] O'Connor AM (1995). Validation of a decisional conflict scale. Med Decis Mak.

[33] Ferron Parayre A, Labrecque M, Rousseau M, Turcotte S, Legare F (2014). Validation of SURE, a four-item clinical checklist for detecting decisional conflict in patients. Med Decis Mak.

[34] Strull WM, Lo B, Charles G (1984). Do patients want to participate in medical decision making?. JAMA.

[35] Degner LF, Sloan JA (1992). Decision making during serious illness: what role do patients really want to play?. J Clin Epidemiol.

[36] Melbourne E, Sinclair K, Durand M-A, Légaré F, Elwyn G (2010). Developing a dyadic OPTION scale to measure perceptions of shared decision making. Patient Educ Couns.

[37] Melbourne E, Roberts S, Durand M-A, Newcombe R, Légaré F, Dyadic EG, OPTION (2011). Measuring perceptions of shared decision-making in practice. Patient Educ Couns.

[38] Kriston L, Scholl I, Hölzel L, Simon D, Loh A, Härter M (2010). The 9-item shared decision making questionnaire (SDM-Q-9). Development and psychometric properties in a primary care sample. Patient Educ Couns.

[39] Shrive FM, Stuart H, Quan H, Ghali WA (2006). Dealing with missing data in a multi-question depression scale: a comparison of imputation methods. BMC Med Res Methodol.

[40] Thompson-Leduc P, Turcotte S, Labrecque M, Legare F (2016). Prevalence of clinically significant decisional conflict: an analysis of five studies on decision-making in primary care. BMJ Open.

[41] Gallagher D, Ni Mhaolain A, Crosby L, Ryan D, Lacey L, Coen RF (2011). Self-efficacy for managing dementia may protect against burden and depression in Alzheimer's caregivers. Aging Ment Health.

[42] Mohamed S, Rosenheck R, Lyketsos CG, Schneider LS (2010). Caregiver burden in Alzheimer disease: cross-sectional and longitudinal patient correlates. Am J Geriatr Psychiatry.

[43] Yeager CA, Hyer LA, Hobbs B, Coyne AC (2010). Alzheimer's disease and vascular dementia: the complex relationship between diagnosis and caregiver burden. Issues Ment Health Nurs.

[44] Zawadzki L, Mondon K, Peru N, Hommet C, Constans T, Gaillard P (2011). Attitudes towards Alzheimer's disease as a risk factor for caregiver burden. Int Psychogeriatr.

[45] Davis JD, Tremont G (2007). Impact of frontal systems behavioral functioning in dementia on caregiver burden. J Neuropsychiatr Clin Neurosci.

[46] Collins C, Jones R (1997). Emotional distress and morbidity in dementia carers: a matched comparison of husbands and wives. Int J Geriatr Psychiatry.

[47] Bedard M, Kuzik R, Chambers L, Molloy DW, Dubois S, Lever JA (2005). Understanding burden differences between men and women caregivers: the contribution of care-recipient problem behaviors. Int Psychogeriatr.

[48] Elwyn G, Frosch DL, Kobrin S (2016). Implementing shared decision-making: consider all the consequences. Implement Sci.

[49] Winter L, Moss MS, Hoffman C (2009). Affective forecasting and advance care planning: anticipating quality of life in future health statuses. J Health Psychol.

[50] Charles C, Gafni A, Whelan T (1997). Shared decision-making in the medical encounter: what does it mean? (or it takes at least two to tango). Soc Sci Med.

