# Correction to: Time to move? Factors associated with burden of care among informal caregivers of cognitively impaired older people facing housing decisions: secondary analysis of a cluster randomized trial

**DOI:** 10.1186/s12877-020-1437-z

**Published:** 2020-02-05

**Authors:** Alexandrine Boucher, Julie Haesebaert, Adriana Freitas, Rhéda Adekpedjou, Marjolaine Landry, Henriette Bourassa, Dawn Stacey, Jordie Croteau, Geneviève Painchaud Guérard, France Légaré

**Affiliations:** 1 Canada Research Chair in Shared Decision Making and Knowledge Translation, and Population Health and Practice-Changing Research Group, Université Laval Primary Care Research Centre (CERSSPL-UL), Quebec, Canada; 2 Department of Family Medicine and Emergency Medicine, Faculty of Medicine, Université Laval, Quebec, Canada; 3 Department of Nursing of Université du Québec à Trois-Rivières, Quebec, Canada; 4 Caregiver Partner, Quebec, Canada; 5 Ottawa Hospital Research Institute, Ottawa, Canada; 6 School of Nursing, University of Ottawa, Ottawa, Canada; 7 Centre intégré universitaire de santé et services sociaux (CIUSSS) de la Capitale-Nationale, Pavillon Landry-Poulin, entrée A-1-2, bureau A-4574, 2525, chemin de la Canardière, Quebec, QC G1J 0A4 Canada

**Correction to: BMC Geriatrics**

**https://doi.org/10.1186/s12877-019-1249-1**

Following publication of the original article [1], we have been notified that one of the authors’ given name and last names are reversed and misspelled and thus not reflected correctly (given name now is Painchaud-Guérard and it should be Geneviève and last name now is Geneviève and it should be Painchaud Guérard).

The given name and last name of the author should be reflected as follows:

Geneviève Painchaud Guérard
